# A novel dual elastography-based model for screening high-risk varices in hepatitis B virus-related cirrhosis

**DOI:** 10.3389/fmed.2026.1707998

**Published:** 2026-02-24

**Authors:** Xing Hu, Min Chen, Ying Zheng, Xiuhua Yang, Wei Zhang, Yao Zhang, Zhiyong Yin, Jintang Liao, Yangshuo Tang, Jie Yu, Ping Liang, Fankun Meng

**Affiliations:** 1 Center of Ultrasound and Functional Diagnosis, Beijing Youan Hospital, Capital Medical University, Beijing, China; 2 Department of Ultrasound, Fifth Medical Center of Chinese PLA General Hospital, Beijing, China; 3 Department of Interventional Ultrasound, The First Affiliated Hospital of Harbin Medical University, Harbin, Heilongjiang, China; 4 Department of Ultrasound, Beijing Ditan Hospital, Capital Medical University, Beijing, China; 5 Department of Ultrasound, Xiangya Hospital, Central South University, Changsha, Hunan, China; 6 Department of Interventional Ultrasound, Fifth Medical Center of Chinese PLA General Hospital, Beijing, China

**Keywords:** cirrhosis, dual elastography, hepatitis B, high-risk varices, non-invasive test

## Abstract

**Background and objective:**

Variceal bleeding carries high mortality and recurrence rates, underscoring the urgent need for effective non-invasive tests (NITs) to assess the severity of esophageal varices (EVs) and predict bleeding risk. This study aimed to develop and validate a robust model for safely excluding high-risk varices (HRVs) in patients with hepatitis B virus (HBV)-related cirrhosis and to compare its diagnostic performance and clinical utility with established NITs.

**Methods:**

Consecutive patients with HBV-related cirrhosis were prospectively recruited from five centers and underwent dual elastography (dual-elasto) within 1 week of esophagogastroduodenoscopy (EGD). A training cohort from four centers was used to identify predictive variables for HRVs, which were analyzed using machine learning to develop the most reliable model. The final model was externally validated in an independent cohort from the fifth center. Diagnostic efficacy and clinical utility were compared across multiple NITs, including the Baveno VI criteria (B6C), expanded Baveno VI criteria (EB6C), platelet–spleen ratio (PSR), and RESIST-HCV criteria (RESIST).

**Results:**

Among 703 screened patients, 328 were enrolled (training cohort *n* = 184; validation cohort *n* = 144). Using logistic regression, we developed the DELU model incorporating six variables: platelet count (PLT), alanine aminotransferase (ALT), ascites, portal vein thrombosis (PVT), inverse difference moment (IDM), and liver stiffness (liver Vs). DELU achieved areas under the receiver operating characteristic curve (AUROCs) of 0.822 (training) and 0.779 (validation), outperforming RESIST (0.600 and 0.626) and B6C (0.569 and 0.575). EB6C and PSR were excluded because they exceeded the 5% missed-HRV threshold. DELU demonstrated the highest spared-EGD rates (20.7% training; 11.1% validation) and maintained robust performance across subgroups, including Child-Pugh B/C (AUROC 80.3%), Child-Pugh A (79.7%), antiviral therapy (ART)-treated (82.5%), and virologically suppressed patients (84.6%). Performance was not influenced by hepatocellular carcinoma (HCC) status, sex, or body mass index (BMI).

**Conclusion:**

DELU is a reliable and safe non-invasive tool for excluding HRVs in HBV-related cirrhosis, providing superior diagnostic accuracy and significant endoscopy-sparing potential across diverse clinical settings. RESIST may serve as a safe alternative when elastography is unavailable.

**Clinical trial registration:**

[ClinicalTrials.gov], identifier [NCT04640350].

## Introduction

Variceal bleeding is a common and life-threatening complication of cirrhosis, characterized by abrupt onset and a 6-week mortality rate of 15–25% (1). Given its high mortality and recurrence rates, current guidelines emphasize the importance of early detection, risk stratification, and continuous monitoring of high-risk varices (HRVs) (2, 3). Although esophagogastroduodenoscopy (EGD) remains the gold standard for evaluating esophageal varices (EVs), its invasive nature, limited tolerability, and impracticality for routine surveillance highlight the urgent need for reliable non-invasive tests (NITs) to assess EV severity and predict variceal bleeding risk.

Several NITs have been proposed, such as the platelet–spleen ratio (PSR) (4) and the RESIST-HCV criteria (RESIST) (5), which have shown diagnostic potential but lack adequate validation in patients with hepatitis B virus (HBV)-related cirrhosis. Current guidelines recommend combining platelet count (PLT) with liver stiffness measurement (LSM) obtained via transient elastography (TE) to evaluate portal hypertension (PH) and reduce unnecessary EGDs (6, 7). The Baveno criteria were primarily designed for asymptomatic compensated advanced chronic liver disease (cACLD) to identify patients who can safely avoid screening endoscopy. Although the Baveno VI criteria (B6C) have been widely adopted in chronic liver disease (CLD) (8), their EGD-sparing rates remain suboptimal, and the safety of the expanded Baveno VI criteria (EB6C) still requires further validation (9).

Moreover, TE, as a shear-wave elastography (SWE) technique, is influenced by hepatic inflammation, cholestasis, and increased intrahepatic blood flow, limiting its accuracy in assessing HRVs because it does not fully reflect fibrosis progression (10). Dual-elastography (dual-elasto), which integrates SWE with strain elastography (SE), offers complementary information and can overcome the limitations of SWE in the setting of hepatic inflammation. By jointly assessing fibrosis-related stiffness and heterogeneity, dual-elasto may enhance non-invasive evaluation of EVs (11, 12); to our knowledge, this approach has not been previously applied to HRVs stratification.

Therefore, the present study aimed to (1) develop and validate a novel dual-elasto-based risk prediction model for safely excluding HRVs in a broad population of patients with HBV-related cirrhosis, and (2) compare its diagnostic accuracy with established NITs, while assessing the impact of clinical factors such as liver disease severity and antiviral therapy (ART) use on model performance and applicability.

## Materials and methods

### Patients and study design

This prospective, multicenter clinical trial consecutively enrolled patients with HBV-related cirrhosis from five centers between January 2022 and June 2025. According to the approved study protocol (ClinicalTrials.gov: NCT04640350), all participants underwent EGD and dual-elasto of the liver at enrollment. The study protocol was approved by the Institutional Review Boards of all participating centers and was conducted in accordance with the Declaration of Helsinki. Written informed consent was obtained from all participants. The inclusion criteria were: (1) age between 18 and 75 years; (2) diagnosis of HBV-related cirrhosis based on clinical, radiological, or histopathological findings; and (3) dual-elasto performed within 1 week of EGD. The exclusion criteria included: (1) history of variceal bleeding; (2) use of nonselective β-blockers; (3) prior interventional therapy (e.g., trans-jugular intrahepatic portosystemic shunt, variceal ligation, or sclerotherapy); (4) non-cirrhotic portal hypertension; (5) large-volume ascites; and (6) unreliable dual-elasto measurements. Patients were stratified and analyzed according to sex, body mass index (BMI), Child-Pugh classification, ART status, viral suppression after ART, and the presence of primary hepatocellular carcinoma (HCC).

### EGD

Upper gastrointestinal endoscopy was performed by two experienced endoscopists who were blinded to all clinical and elastography data. Findings were recorded in a standardized format, and EVs were graded according to international guidelines (6). HRVs were defined as EVs with grade 1 and red signs, grade 2, or grade 3, all of which require clinical intervention.

### Clinical and abdominal ultrasonographic data

Demographic and clinical data, including age, sex, BMI, medical history, cirrhosis-related complications, and ART status (both treatment status and viral suppression), were collected for all patients. Viral suppression was defined as undetectable or below-lower-limit HBV-DNA levels for at least 6 months prior to enrollment. Laboratory tests were performed within 1 week of EGD, and standardized abdominal ultrasonography was conducted within the same timeframe.

### Dual elastography measurements

All operators were radiologists with over 5 years of experience in abdominal ultrasonography and had completed training with more than 50 dual-elasto cases before study initiation. All radiologists were blinded to EGD results. Participants fasted for at least 12 h prior to examination. Dual-elasto was performed using an ultrasound system (Arietta 850; FujiFilm Healthcare, Tokyo, Japan) equipped with a C715 probe (1–6 MHz) under elasticity-specific settings. Participants were examined in the supine position with the right arm fully abducted. Liver segments S5 or S8 were selected, and the region of interest (ROI; 3 × 2.5 cm^2^) was placed 1–2 cm below the hepatic capsule, avoiding major vessels. Measurements were performed during breath-holding at the end of normal expiration (see Supplementary Figure 1 for details). Five consecutive dual-elasto images were acquired, and the median value was recorded. Twelve raw elasticity parameters were extracted: (1) MEAN (mean relative strain value within the ROI); (2) SD (standard deviation); (3) AREA (low-strain area); (4) COMP (complexity of the low-strain area); (5) KURT (kurtosis); (6) SKEW (skewness); (7) CONT (contrast); (8) ENT (entropy); (9) IDM (inverse difference moment); (10) ASM (angular second moment); (11) CORR (correlation); and (12) liver Vs (shear-wave velocity in SWE). The first 11 parameters were derived from strain elastography (SE), whereas liver Vs was obtained from shear-wave elastography (SWE). Measurements were considered valid if the net effective shear-wave velocity (VsN) was ≥ 60% and each SE image displayed a stable strain curve with a continuous cardiac cycle. All dual-elasto examinations were performed within 1 week of EGD.

### Non-invasive tests evaluated

In this study, the following previously established NITs were evaluated: B6C: LSM < 20 kPa and PLT > 150 × 10^9^/L (6). EB6C: LSM < 25 kPa and PLT > 110 × 10^9^/L (7). RESIST: serum albumin > 3.6 g/dL and PLT > 120 × 10^9^/L (5). PSR: PLT ( × 10^3^/μL) divided by spleen bipolar diameter (mm), as originally proposed by Giannini et al. (4).

### Statistical analysis

Quantitative variables were summarized as means ± standard deviation (SD) or medians with interquartile ranges (IQRs) and compared using Student’s *t-*test or the Manniation (SD) or meas appropriate. Categorical variables were expressed as counts (percentages) and analyzed using the chi-squared or Fisher’s exact test. Missing data accounted for < 5% of all variables. Given the low proportion of missingness, missing values in the primary analysis were handled using simple imputation methods (mean or median for continuous variables and mode for categorical variables), which is unlikely to materially affect model performance.

Significant predictors of HRVs were identified using the least absolute shrinkage and selection operator (LASSO) method, while linear regression explored associations between dual-elasto parameters and EVs grading. Statistically significant variables were incorporated into multiple machine learning (ML) models, including logistic regression (LR), k-nearest neighbors (KNN), support vector machines (SVM), Gaussian naive Bayes (GNB), decision trees (DT), random forests (RF), and gradient boosting decision trees (GBDT). Model performance was assessed using the area under the receiver operating characteristic curve (AUROC), sensitivity, specificity, positive predictive value (PPV), and negative predictive value (NPV). AUROCs were compared using the DeLong test. PLT counts were capped at 150 × 10^9^/L, as the risk of portal hypertension and varices plateaus beyond this threshold (13). The most accurate and stable model was selected based on the Akaike information criterion (AIC) and AUROC values. Calibration was evaluated using calibration curves, clinical utility via decision curve analysis (DCA), and nomograms were developed from the final model. Decision thresholds were selected using a predefined two-step approach, combining Youden index–based ROC analysis in the training cohort with a clinical safety constraint (missed-HRVs rate < 5%), and were subsequently applied to the validation cohort without further optimization. All analyses were performed using R software (version 4.3.0; Vienna, Austria) and SPSS (version 25.0; IBM Corp., United States). A two-sided *p* < 0.05 was considered statistically significant.

## Results

### Patient characteristics

**FIGURE 1:**
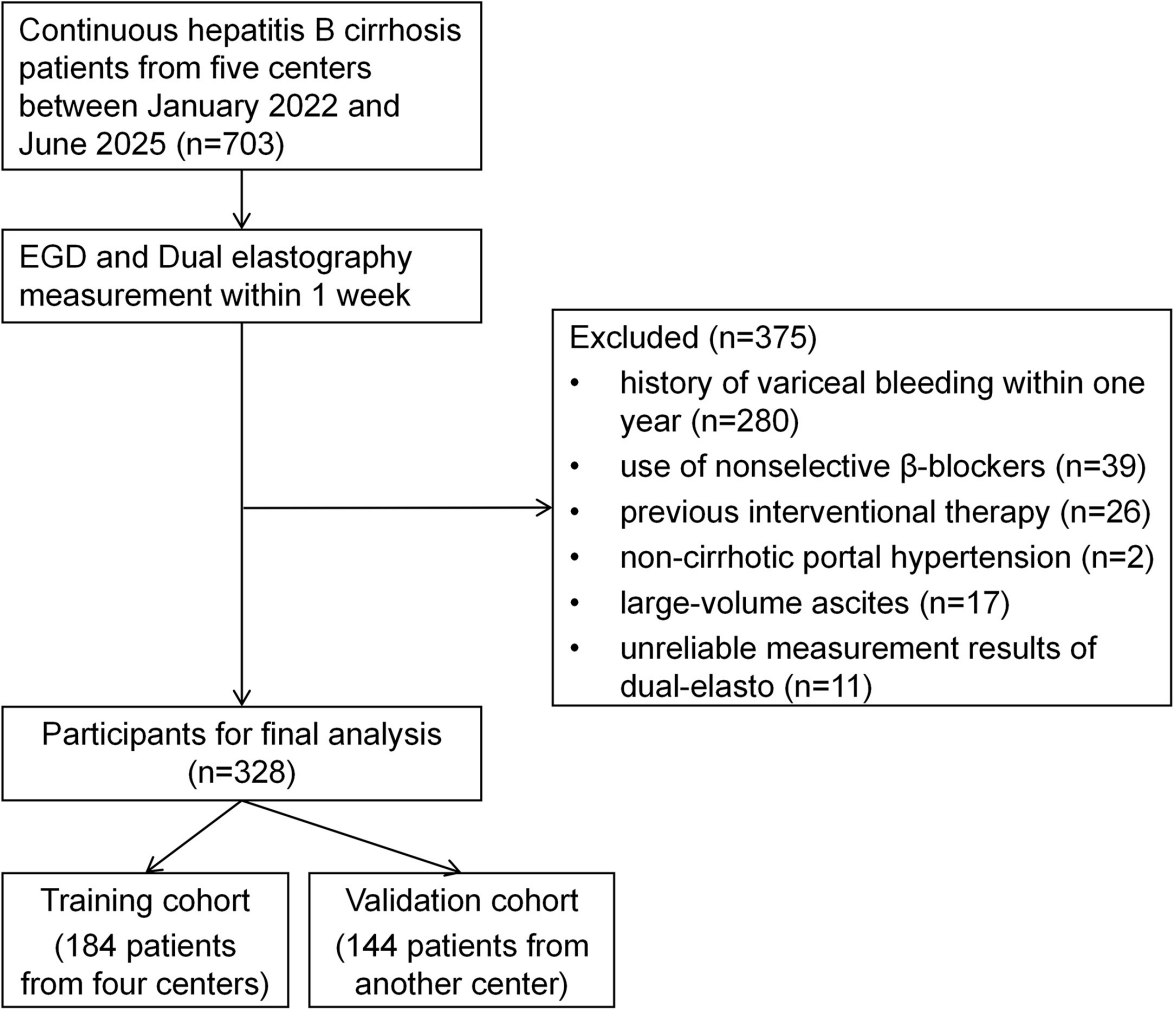
Flowchart of patient enrollment and study design.

A total of 703 patients were screened, and 375 were excluded based on the exclusion criteria. Ultimately, 328 patients with HBV-related cirrhosis were included. Four centers contributed 184 patients to the training cohort, and another center contributed 144 patients to the validation cohort (Figure 1).

In the training cohort, most patients had compensated cirrhosis, with 143 (77.7%) classified as Child-Pugh A. A total of 155 patients (84.2%) were receiving ART, and 86 (55.5%) of them achieved viral suppression. EVs and HRVs were detected in 156 (84.8%) and 90 (48.9%) patients, respectively. In the validation cohort, 131 patients (91.0%) were Child-Pugh A. Sixty-six patients (45.8%) received ART, of whom 46 (69.7%) achieved viral suppression. EVs and HRVs were identified in 127 (88.2%) and 100 (69.4%) patients, respectively (Table 1).

**TABLE 1:** Baseline characteristics of patients in the training and validation cohorts.

Variable	Total population (*n* = 328)	Training cohort (*n* = 184)	Validation cohort (*n* = 144)	*P*
Age (year)	53.1 ± 10.3	53.3 ± 10.4	52.9 ± 10.2	0.750
Male, n (%)	234 (71.3)	129 (70.1)	105 (72.9)	0.620
BMI (kg/m^2^)	23.7 (21.7–26.3)	24.3 (21.8–27.0)	23.2 (21.5–24.6)	0.004
**History of disease, n (%)**				
HCC	89 (27.1)	55 (29.9)	34 (23.6)	0.210
Hypertension	42 (12.8)	33 (17.9)	9 (6.3)	0.002
Diabetes	38 (11.6)	30 (16.3)	8 (5.6)	0.003
**Antiviral treatment, n (%)**				
Anti-HBV treatment	221 (67.4)	155 (84.2)	66 (45.8)	<0.001
Virally suppressed	132 (59.7)	86 (55.5)	46 (69.7)	0.049
Child-pugh class, n (%)				0.005
Class A	274 (83.5)	143 (77.7)	131 (91.0)	
Class B	32 (9.8)	24 (13.0)	8 (5.6)	
Class C	22 (6.7)	17 (9.2)	5 (3.5)	
Esophageal varices, n (%)				0.001
No varices	45 (13.7)	28 (15.2)	17 (11.8)	
Grade 1	93 (28.4)	66 (35.9)	27 (18.8)	
Grade 2	67 (20.4)	29 (15.8)	38 (26.4)	
Grade 3	123 (37.5)	61 (33.1)	62 (43.1)	
HRVs, n (%)	190 (57.9)	90 (48.9)	100 (69.4)	<0.001
**Laboratory test values**				
WBC ( × 10^9^/L)	3.4 (2.5–4.7)	3.8 (2.7–5.1)	3.0 (2.1–3.9)	<0.001
NEUT ( × 10^9^/L)	1.9 (1.4–2.8)	2.2 (1.4–3.2)	1.7 (1.3–2.4)	<0.001
PLT ( × 10^9^/L)	69.0 (49.3–105.0)	76.0 (51.5–113.0)	60.0 (44.0– 95.0)	0.005
TP (g/L)	63.6 ± 7.7	64.6 ± 8.0	62.2 ± 7.0	0.004
ALB (g/L)	34.8 (30.0–39.5)	33.8 (30.0–39.0)	36.0 (31.0–40.0)	0.150
TBIL (μmoL/L)	27.5 (17.7–46.6)	35.2 (19.7–58.3)	23.0 (15.5–32.7)	<0.001
DBIL (μmoL/L)	10.8 (6.2–20.4)	14.9 (6.8–25.3)	8.8 (5.8–13.7)	<0.001
ALT (U/L)	26.0 (18.0–41.0)	26.5 (20.0–41.5)	25.0 (17.0–39.0)	0.069
AST (U/L)	36.0 (26.3–55.0)	40.0 (29.0–61.3)	31.5 (24.0–48.0)	<0.001
GGT (U/L)	35.0 (20.0–78.5)	40.5 (21.0–104.0)	28.5 (18.5–62.0)	0.057
ALP (U/L)	96.5 (74.3–137.8)	103.0 (79.0–153.5)	89.0 (67.0–118.0)	<0.001
SCr (μmoL/L)	66.0 (56.0–77.8)	60.0 (51.0–70.5)	73.50 (65.0–83.0)	<0.001
PT (s)	14.2 (12.8–16.4)	14.5 (12.7–17.3)	14.0 (12.9–15.6)	0.150
PTA (%)	68.4 ± 19.9	68.7 ± 20.2	68.0 ± 19.7	0.780
INR	1.25 (1.13–1.46)	1.27 (1.13–1.52)	1.24 (1.13–1.39)	0.087
HBsAg (col)	2,062 (703–3,298)	1,893 (683–2,395)	3,713 (874–5897)	<0.001
**Ultrasonic measurements**				
Spleen diameter (mm)	144.8 ± 32.6	141.6 ± 32.8	148.8 ± 32.0	0.046
Spleen vein diameter (mm)	8.6 (7.1–10.6)	8.2 (7.0–9.9)	9.4 (7.5–11.8)	0.001
Spleen vein Vm (cm/s)	17.9 (14.9–21.7)	18.9 (16.0–22.3)	16.6 (13.9–19.7)	<0.001
Portal vein diameter (mm)	12.0 (10.8–13.4)	12.3 (10.9–13.4)	11.8 (10.8–13.5)	0.190
Portal vein Vm (cm/s)	17.1 (13.9–21.2)	18.4 (14.4–22.6)	15.9 (13.2–19.2)	<0.001
**Complications of cirrhosis, n (%)**				
PVT, n (%)	55 (16.8)	35 (19.0)	22 (15.3)	0.460
Collateral circulation, n (%)	232 (70.7)	160 (87.0)	72 (50.0)	<0.001
Ascites, n (%)	98 (29.9)	83 (45.1)	15 (10.4)	<0.001

BMI, Body mass index; HCC, Hepatocellular carcinoma; HBV, Hepatitis B virus; HRVs, High-risk varices; WBC, white blood cell count; NEUT, neutrophil count; PLT, platelet count; TP, total protein; ALB, albumin; TBIL, total bilirubin; DBIL, direct bilirubin; ALT, alanine aminotransferase; AST, aspartate aminotransferase; GGT, γ-Glutamyl transpeptidase; ALP, Alkaline phosphatase; SCr, Serum creatinine; PT, Prothrombin time; PTA, Prothrombin activity; INR, international normalized ratio; Vm, mean velocity; PVT, Portal vein thrombosis.

### Construction and validation of the DELU model

**FIGURE 2:**
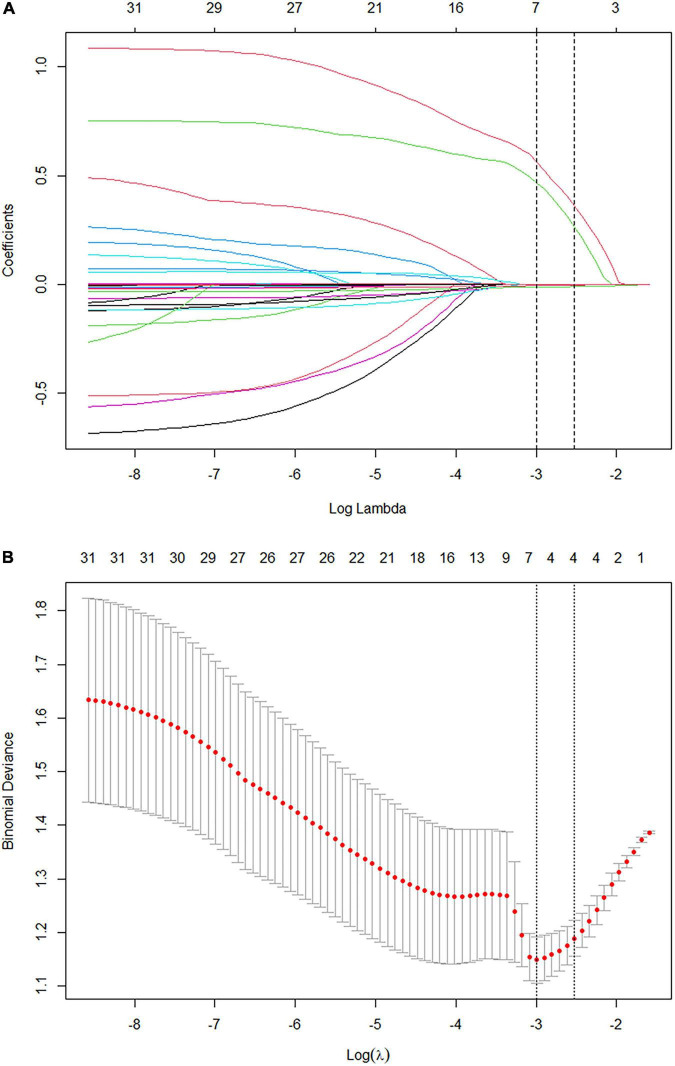
Selection of predictors for high-risk varices using least absolute shrinkage and selection operator (LASSO) regression. **(A)** Coefficient paths of 31 candidate variables in the training cohort, with the optimal penalty parameter (λ) selected by 10-fold cross-validation (vertical dashed line), retaining four variables. **(B)** Determination of the optimal λ by 10-fold cross-validation, with the dotted line indicating the minimum cross-validated error.

LASSO regression in the training cohort identified four key predictors of HRVs: PLT, alanine aminotransferase (ALT), ascites, and portal vein thrombosis (PVT) (Figure 2). Linear regression further revealed that IDM and liver Vs were significantly associated with EV grading (Supplementary Tables 3, 4). Based on these six parameters (PLT, ALT, ascites, PVT, IDM, liver Vs), three stepwise models were developed: (1) Laboratory metrics only; (2) Laboratory metrics + ultrasound indicators; (3) Laboratory metrics + ultrasound indicators + dual-elasto parameters. Each model was tested across multiple machine learning (ML) algorithms, with diagnostic performance evaluated by the AUROC (Table 2).

**TABLE 2:** AUROCs of different machine learning models for predicting HRVs in the training and validation cohorts.

Algorithm	Model 1	Model 2	Model 3
**Training cohort (*n* = 184)**			
LR	0.775	0.815	0.822
KNN	0.783	0.811	0.853
SVM	0.713	0.721	0.761
GNB	0.725	0.777	0.783
DT	0.907	0.970	1.000
RF	0.767	0.797	0.857
GBDT	0.906	0.969	1.000
**Validation cohort (*n* = 144)**			
LR	0.758	0.782	0.779
KNN	0.728	0.691	0.675
SVM	0.759	0.742	0.760
GNB	0.752	0.786	0.810
DT	0.596	0.610	0.538
RF	0.770	0.797	0.777
GBDT	0.627	0.677	0.647

AUROC, Area Under the Receiver Operating Characteristic curve; HRVs, high-risk varices; LR, logistic regression; KNN, k-Nearest Neighbors; SVM, Support Vector Machine; GNB, Gaussian Naive Bayes classifiers; DT, Decision Tree; RF, Random Forest; GBDT, Gradient Boost Decision Tree; PLT, platelet count; ALT, alanine aminotransferase; PVT, Portal vein thrombosis; IDM, inverse difference moment; Model 1: Laboratory metrics only (PLT + ALT); Model 2: Laboratory metrics + ultrasound indicators (PLT + ALT + ascites + PVT); Model 3: Laboratory metrics + ultrasound indicators + dual-elastography parameters (PLT + ALT + ascites + PVT + IDM + liver Vs).

**FIGURE 3:**
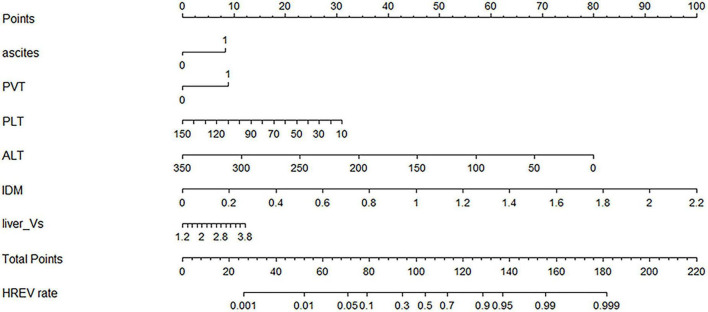
Nomogram of the DELU model for estimating the probability of high-risk varices.

Model 3, incorporating all six parameters within a logistic regression framework, demonstrated the highest accuracy and stability and was designated as the DELU model. Regression coefficients for all models are presented in Supplementary Table 5. A nomogram based on DELU was developed to estimate HRVs risk (Figure 3), achieving C-statistics of 0.822 in the training cohort and 0.779 in the validation cohort. Calibration curves confirmed good model fit (*p* > 0.05) (Supplementary Figure 2), and decision curve analysis (DCA) demonstrated broad clinical utility across varying risk thresholds (Supplementary Figure 3).

### Performance and safety of DELU and other NITs

The EB6C and PSR models were excluded because their missed-HRV rates exceeded 5%. Among the remaining models (DELU, B6C, RESIST), DELU achieved the highest diagnostic accuracy, with AUROCs of 0.822 (training) and 0.779 (validation), outperforming RESIST (0.600, 0.626) and B6C (0.569, 0.575). In terms of spared-EGD rates, DELU again performed best (20.7% training; 11.1% validation), followed by RESIST (13.6%, 9.7%) and B6C (7.1%, 5.6%) (Table 3).

**TABLE 3:** Diagnostic performance of the DELU model and other NITs for predicting HRVs in the training and validation cohorts.

NITs	AUROC (%)	Sensitivity (%)	Specificity (%)	PPV (%)	NPV (%)	Spared EGD (%)	Missed HRV (%)
**Training cohort (*n* = 184)**							
DELU (cutoff 0.16)	82.2	97.8	38.3	60.3	94.7	20.7	2.2
Baveno VI	56.9	100	13.8	52.6	100	7.1	0
Expand Baveno VI	62.6	91.1	34.0	56.9	80.0	21.7	8.9
PSR	73.0	91.1	38.3	58.6	81.8	23.9	8.9
RESIST	60.0	96.7	23.4	54.7	88.0	13.6	3.3
**Validation cohort (*n* = 144)**							
DELU (cutoff 0.16)	77.9	97.0	29.5	75.8	81.3	11.1	3.0
Baveno VI	57.5	99.0	15.9	72.8	87.5	5.6	1.0
Expand Baveno VI	63.5	93.0	34.1	76.2	68.2	15.3	7.0
PSR	76.8	94.0	36.4	77.0	72.7	15.3	6.0
RESIST	62.6	98.0	27.3	75.4	85.7	9.7	2.0

NITs, noninvasive tests; HRVs, high-risk varices; AUROC, Area Under the Receiver Operating Characteristic curve; PPV, positive predictive value; NPV, negative predictive value; EGD, Esophagogastroduodenoscopy.

### Diagnostic efficacy of NITs in child-pugh subgroups

As performance was consistent between cohorts, pooled results are presented. Pooled analysis showed DELU achieved significantly higher AUROCs than B6C and RESIST in both Child-Pugh A and B/C subgroups (*p* < 0.05), with AUROCs of 80.3% (B/C) and 79.7% (A). In contrast, B6C and RESIST performed poorly in the B/C subgroup (AUROCs < 55%) (Table 4).

**TABLE 4:** Performance of NITs for predicting HRVs across child-pugh subgroups in the overall patient population.

NITs	AUROC (%)		Spared EGD (%)		Missed HRV (%)	
	Child A (*n* = 274)	Child B + C (*n* = 54)	Child A (*n* = 274)	Child B + C (*n* = 54)	Child A (*n* = 274)	Child B + C (*n* = 54)
DELU	79.7	80.3	16.4	16.7	2.5	3.4
Baveno VI	57.6	54.2	6.9	3.7	0.6	0
RESIST	62.5	54.2	13.5	3.7	3.1	0

NITs, noninvasive tests; HRVs, high-risk varices; AUROC, Area Under the Receiver Operating Characteristic curve; EGD, Esophagogastroduodenoscopy.

Spared-EGD rates were highest with DELU in both subgroups (16.4% A; 16.7% B/C), whereas B6C and RESIST achieved markedly lower rates in the B/C subgroup (3.7, 3.7%) compared with the A subgroup (6.9, 13.5%; *p* < 0.05). Notably, DELU spared-EGD rates did not differ significantly between the Child-Pugh A and B/C subgroups (16.4% vs. 16.7%, *p* > 0.05).

### Diagnostic efficacy of NITs in ART, naive-ART, and other subgroups

DELU achieved significantly higher AUROCs than B6C and RESIST in both ART-treated (82.5%) and naive-ART (74.3%) subgroups (*p* < 0.05). Spared-EGD rates were also highest with DELU (18.6%), followed by RESIST (11.8%) and B6C (6.3%) (Table 5).

**TABLE 5:** Performance of NITs for predicting HRVs across ART-treated and ART-naïve subgroups in the overall patient population.

NITs	AUROC (%)		Spared EGD (%)		Missed HRV (%)	
	ART (*n* = 221)	Naive-ART (*n* = 107)	ART (*n* = 221)	Naive-ART (*n* = 107)	ART (*n* = 221)	Naive-ART (*n* = 107)
DELU	82.5	74.3	18.6	12.1	1.8	3.8
Baveno VI	56.4	60.1	6.3	6.5	0	1.3
RESIST	60.0	66.0	11.8	12.1	1.8	3.8

NITs, noninvasive tests; HRVs, high-risk varices; AUROC, Area Under the Receiver Operating Characteristic curve; EGD, Esophagogastroduodenoscopy; ART, antiviral therapy.

Across subgroups defined by viral suppression, HCC, sex, and BMI, the DELU model consistently outperformed other models (*p* < 0.05), achieving an AUROC of 84.6% in the viral suppression subgroup. When stratified by HCC status, sex, and BMI, the diagnostic performance of DELU and other NITs remained consistent, with no statistically significant differences detected across these subgroups (all *p* > 0.05) (details in Supplementary Tables 6–13).

## Discussion

Endoscopy remains the gold standard for evaluating esophageal varices; however, its invasive nature and limited patient compliance restrict its widespread application. Consequently, reliable non-invasive methods for identifying low-risk patients and minimizing unnecessary endoscopies are of significant clinical importance. In this study, we developed and validated a novel dual elastography-based model (DELU), which demonstrated excellent discriminatory power for excluding high-risk varices (HRVs) in patients with HBV-related cirrhosis. To our knowledge, this is the first multicenter, prospective study to integrate SE–derived heterogeneity metrics with SWE–derived liver stiffness for predicting HRVs. Furthermore, the DELU model was developed and externally validated in a relatively large cohort of patients with HBV-related cirrhosis and demonstrated stable performance across several clinically challenging subgroups.

Non-invasive predictors of esophageal varices and variceal bleeding have been widely studied; however, single serological or imaging indicators are often inadequate for accurate risk assessment. Multiparametric integration offers greater predictive power (14). We developed the DELU model integrating laboratory data, ultrasonography, and dual elastography, incorporating six parameters: PLT, ALT, ascites, PVT, IDM, and liver Vs. Each component contributes distinct value. Thrombocytopenia reflects hypersplenism and the severity of portal hypertension, and its combination with liver stiffness improves the safety of HRV exclusion (15, 16). ALT reflects inflammatory activity and corrects inflammation-related bias in stiffness measurements (12). Ascites, a typical manifestation of decompensation due to portal hypertension, is strongly associated with increased risk of esophageal variceal bleeding (17). PVT elevates portal pressure through venous obstruction, promoting variceal progression and bleeding, and serves as a high-risk prognostic signal (18). Including ascites and PVT enables DELU to capture “high-risk phenotypes” within heterogeneous real-world populations. Elastography-derived measures further strengthen the model. Liver Vs directly quantifies hepatic stiffness and reflects portal hypertension, while IDM characterizes tissue texture homogeneity, capturing microstructural features beyond stiffness. To construct the model, we compared logistic regression with several machine learning approaches. Logistic regression ultimately provided robust and consistent efficacy across both training and validation cohorts, whereas other machine learning models, despite good performance in training, showed reduced efficacy in validation—likely due to overfitting (19).

The risk and severity of EV bleeding increase substantially with the progression of CLD (20). Therefore, an ideal NIT should maintain a missed HRV rate below 5% while ensuring a high rate of spared EGD. In this study, DELU, B6C, and RESIST all met the criterion of a missed HRV rate below 5% across various patient populations. Overall, DELU demonstrated the highest AUROC and spared EGD rates, while B6C showed the lowest performance among the three models. Consistent with previous studies, our findings suggest that B6C is both robust and safe (16, 21). However, the spared EGD rate of B6C was notably low in our cohort, indicating its relatively limited clinical utility. In contrast, although EB6C showed a higher spared EGD rate, it was excluded because its missed HRV rate exceeded the safety threshold, a finding consistent with previous studies (22). Because TE is often not feasible in clinical practice, we also evaluated the diagnostic efficacy of PSR and RESIST as alternative tests to avoid endoscopy. PSR was excluded from our study because its missed HRV rate exceeded 5%. Currently, most studies on PSR are based on small-scale clinical trials, raising concerns about its clinical utility (23). Additionally, our findings suggest that RESIST is both safe and effective for excluding HRV, with a spared EGD rate second only to DELU. These results support the use of RESIST as an alternative when TE or dual-elasto is unavailable.

The prevalence of HRV, PLT, and LSM is closely associated with the severity of CLD (24), which reduces the diagnostic efficacy of NITs in advanced stages (16). The Baveno VI Consensus recommends limiting the B6C model to compensated disease, and evidence for its use in advanced HBV-related cirrhosis remains limited. Our subgroup analyses confirmed that while B6C maintained safety in HRVs exclusion, its clinical utility was poor due to a low spared EGD rate, particularly in Child-Pugh B/C patients. RESIST showed moderate safety and applicability across Child-Pugh classes, but its spared EGD rate was also limited in advanced disease. In contrast, DELU demonstrated superior performance and was the only NIT with a spared EGD rate exceeding 15% in the Child-Pugh B/C subgroup, indicating clear advantages in severe CLD. Furthermore, as more patients with viral cirrhosis now receive antiviral therapy (ART) and achieve viral suppression (25, 26), the role of NITs in this population is increasingly important. Yet, most previous validations of B6C excluded ART-treated patients (27), and few studies have assessed NITs in this context (21, 28). In our study, DELU, B6C, and RESIST were all safe in ART-treated and ART-naive patients, with DELU showing the highest discriminatory power in both groups.

Although the DELU model was developed in patients with HBV-related cirrhosis, its constituent variables reflect pathophysiological mechanisms shared across different cirrhosis etiologies. Thrombocytopenia reflects PH, while elastography-derived stiffness and heterogeneity characterize fibrosis and tissue remodeling common to viral, metabolic, and alcohol-related liver diseases. Nevertheless, etiology-specific factors may influence elastography measurements and platelet levels; therefore, the present findings should be interpreted within the context of HBV-related cirrhosis, and application to other etiologies warrants cautious evaluation and external validation. From a clinical implementation perspective, DELU relies on variables that are routinely available in standard hepatology practice. Dual-elastography can be performed within a few minutes using commercially available ultrasound systems without additional preparation beyond routine fasting. With appropriate operator training and adherence to predefined quality criteria, DELU may serve as a non-invasive triage tool to identify patients at low risk for HRVs who may safely defer screening endoscopy, while patients classified as high risk should be referred for endoscopic evaluation.

Beyond the NITs benchmarked in our cohort, several models with similar objectives have been reported. TE-based composite scores such as LSPS integrate liver stiffness with surrogates of PH and can achieve good discrimination, but they primarily reflect macroscopic stiffness with limited assessment of tissue heterogeneity (29). Laboratory-based machine-learning models prioritize accessibility without elastography requirements, although structural liver changes are captured indirectly (17). Imaging-based radiomics models may achieve high AUCs; however, their reliance on contrast-enhanced computed tomography and complex feature-extraction workflows may limit routine clinical implementation (30). In contrast, the DELU model integrates routinely available clinical variables with dual-elasto, combining SWE-derived stiffness and SE-derived heterogeneity to provide complementary structural information while remaining compatible with standard ultrasound workflows.

This study has several limitations. First, the training cohort included patients from four centers across different geographic regions, resulting in inevitable data heterogeneity. To mitigate this, we implemented a standardized protocol and quality control measures before study initiation and provided uniform training for physicians performing dual-elasto assessments to minimize potential selection bias. Second, the spared EGD rates for NITs in our study were generally lower than those reported previously, likely due to the higher HRV prevalence in our population, as the spared EGD rate is closely correlated with HRV prevalence (27, 28). Third, we did not evaluate the efficacy and robustness of NITs across different treatment modalities or durations because of treatment diversity and inconsistent record-keeping.

In conclusion, this multicenter prospective study developed and validated the DELU model, a dual elastography–raphycnon-invasive tool for safely excluding HRVs in patients with hepatitis B cirrhosis. DELU consistently demonstrated superior discriminatory accuracy and higher spared EGD rates compared with established models across diverse clinical scenarios, including advanced CLD and ART-treated populations. When elastography is unavailable, the RESIST model may serve as a safe alternative for HRV exclusion.

## Data Availability

The raw data supporting the conclusions of this article will be made available by the authors, without undue reservation.
