# Supplementary material for: A novel dual elastography-based model for screening high-risk varices in hepatitis B virus-related cirrhosis
Source: Front Med (Lausanne). 2026 Feb 24;13:1707998. doi: 10.3389/fmed.2026.1707998 (PMC12971904; doi:10.3389/fmed.2026.1707998)
Supplement: Supplementary file 1 [file Data_Sheet_1.docx]

**Supplementary Materials**

**Table S1 Participating departments and radiologists involved in the study**

| **Department** | **Cases (n)** | **Radiologist** |
| --- | --- | --- |
| Beijing Youan Hospital, Capital Medical University. | 158 | Fankun Meng, Xing Hu, Ying Zheng |
| Fifth Medical Center of Chinese PLA General Hospital | 144 | Ping Liang, Jie Yu，Min Chen |
| The First Affiliated Hospital of Harbin Medical University | 17 | Xiuhua Yang，Wei Zhang |
| Beijing Ditan Hospital Capital Medical University | 5 | Yao Zhang，Zhiyong Yin |
| Xiangya Hospital Central South University | 4 | Jintang Liao，Yangshuo Tang |

**Table S2 Baseline characteristics of patients in the training cohort**

|  | Training cohort  (n=184) | Low-risk varices (n=94) | High-risk varices (n=90) | *P* |
| --- | --- | --- | --- | --- |
| Age (year) | 53.3±10.4 | 53.4±10.9 | 53.2±10.1 | 0.940 |
| Male, n (%) | 129 (70.1) | 67 (71.0) | 62 (69.0) | 0.750 |
| Body mass index (kg/m^2^) | 24.3 (21.8-27.0) | 25.3 (21.8-27.1) | 23.7 (21.6-27.0) | 0.200 |
| History of disease, n (%) |  |  |  |  |
| HCC | 55 (29.9) | 26 (28.0) | 29 (32.0) | 0.520 |
| Hypertension | 33 (17.9) | 19 (20.0) | 14 (16.0) | 0.450 |
| Diabetes | 30 (16.3) | 15 (16.0) | 15 (17.0) | 1.000 |
| Antiviral treatment, n (%) |  |  |  |  |
| Anti-HBV treatment | 155 (84.2) | 77 (82.0) | 78 (87.0) | 0.420 |
| Virally suppressed | 86 (55.5) | 40 (51.9) | 46 (59.0) | 0.110 |
| Child-Pugh class, n (%) |  |  |  | 0.630 |
| Class A | 143 (77.7) | 75 (80.0) | 68 (76.0) |  |
| Class B | 24 (13.0) | 10 (11.0) | 14 (16.0) |  |
| Class C | 17 (9.2) | 9 (10.0) | 8 (9.0) |  |
| Esophageal varices, n (%) |  |  |  | ＜0.001 |
| No varices | 28 (15.2) | 28 (30.0) | 0 (0) |  |
| Grade 1 | 66 (35.9) | 66 (70.0) | 0 (0) |  |
| Grade 2 | 29 (15.8) | 0 (0) | 29 (32.0) |  |
| Grade 3 | 61 (33.1) | 0 (0) | 61 (68.0) |  |
| Laboratory test values |  |  |  |  |
| WBC (× 10^9^/L) | 3.8 (2.7-5.1) | 4.4(3.0-5.7) | 3.3 (2.5-4.4) | ＜0.001 |
| NEUT (× 10^9^/L) | 2.2 (1.4-3.2) | 2.5 (1.5-3.4) | 1.9 (1.4-2.7) | 0.032 |
| PLT (× 10^9^/L) | 76.0 (51.5-113.0) | 101.0 (69.0-143.0) | 59.5 (48.0- 86.0) | ＜0.001 |
| TP (g/L) | 64.6±8.0 | 65.6±7.4 | 63.6±8.6 | 0.095 |
| ALB (g/L) | 33.8 (30.0-39.0) | 35.6 (30.8-41.0) | 32.6 (29.2-35.9) | 0.002 |
| TBIL (μmol/L) | 35.2 (19.7-58.3) | 33.1 (17.5-66.7) | 37.1 (22.3-56.6) | 0.610 |
| DBIL (μmol/L) | 14.9 (6.8-25.3) | 13.8 (6.0-34.8) | 15.3 (8.6-22.6) | 0.600 |
| ALT (U/L) | 26.5 (20.0-41.5) | 29.5 (21.0-61.5) | 25.0 (19.0-35.0) | 0.002 |
| AST (U/L) | 40.0 (29.0-61.3) | 39.5 (27.7-83.8) | 40.0 (29.8-55.8) | 0.584 |
| GGT (U/L) | 40.5 (21.0-104.0) | 46.5 (26.0-139.6) | 32.5 (18.0-74.0) | 0.022 |
| ALP (U/L) | 103.0 (79.0-153.5) | 103.5 (76.0-158.0) | 102.0 (81.0-149.0) | 0.760 |
| SCr (μmol/L) | 60.0 (51.0-70.5) | 58.6 (51.0-68.0) | 62.0 (50.0-74.0) | 0.260 |
| PT (s) | 14.5 (12.7, 17.3) | 13.9 (11.9, 16.8) | 15.2 (13.3, 17.7) | 0.014 |
| PTA (%) | 68.7±20.2 | 72.1±22.2 | 65.1±17.2 | 0.018 |
| INR | 1.27 (1.13-1.52) | 1.23 (1.06-1.50) | 1.30 (1.19-1.56) | 0.017 |
| HBsAg (col) | 1893 (683-2395) | 1925 (675-2381) | 1872 (688-2423) | 0.847 |
| Ultrasonic measurements |  |  |  |  |
| Spleen diameter (mm) | 141.6±32.8 | 133.3±30.4 | 150.2±33.2 | ＜0.001 |
| Spleen vein diameter (mm) | 8.2(7.0-9.9) | 7.9 (6.9-9.0) | 8.5 (7.5-10.3) | 0.003 |
| Spleen vein Vm (cm/s) | 18.9 (16.0-22.3) | 18.4 (15.4-21.4) | 19.5 (16.3-24.9) | 0.042 |
| Portal vein diameter (mm) | 12.3 (10.9-13.4) | 12.2 (10.8-13.1) | 12.5 (11.0-13.7) | 0.180 |
| Portal vein Vm (cm/s) | 18.4 (14.4-22.6) | 18.4 (14.8-23.1) | 18.3 (14.4-21.7) | 0.560 |
| Complications of cirrhosis, n (%) |  |  |  |  |
| PVT, n (%) | 35 (19.0) | 12 (13.0) | 23 (26.0) | 0.038 |
| Collateral circulation, n (%) | 160 (87.0) | 77 (82.0) | 83 (92.0) | 0.049 |
| Ascites, n (%) | 83 (45.1) | 33 (35.0) | 50 (56.0) | 0.007 |

BMI:Body mass index; HCC: Hepatocellular carcinoma; HBV: Hepatitis B virus; WBC, white blood cell count; NEUT, neutrophil count; PLT, platelet count; TP, total protein; ALB, albumin; TBIL, total bilirubin; DBIL, direct bilirubin; ALT, alanine aminotransferase; AST, aspartate aminotransferase; GGT, γ-Glutamyl transpeptidase; ALP, Alkaline phosphatase; SCr, Serum creatinine; PT, Prothrombin time; PTA, Prothrombin activity; INR, international normalized ratio；Vm：mean velocity; PVT: Portal vein thrombosis

**Table S3 Distribution of dual-elastography indices in the training and validation cohorts**

| Elastography indices | Total population (n=328) | Training cohort (n=184) | Validation cohort (n=144) |
| --- | --- | --- | --- |
| MEAN | 92.55±15.89 | 90.17±16.99 | 95.61±13.82 |
| SD | 66.69±8.29 | 67.07±8.99 | 66.19±7.30 |
| AREA | 35.91±12.72 | 37.81±12.84 | 33.47±12.17 |
| COMP | 38.07±32.52 | 40.13±27.08 | 35.45±38.31 |
| KURT | 2.41±0.32 | 2.41±0.28 | 2.40±0.36 |
| SKEW | 0.51±0.31 | 0.52±0.30 | 0.48±0.31 |
| CONT | 378.66±113.05 | 410.62±120.31 | 337.82±87.80 |
| ENT | 3.57±0.21 | 3.56±0.19 | 3.58±0.23 |
| IDM | 0.13±0.12 | 0.15±0.15 | 0.12±0.05 |
| ASM | 0.01±0.02 | 0.01±0.01 | 0.00±0.02 |
| CORR | 0.96±0.01 | 0.95±0.01 | 0.96±0.01 |
| liver Vs | 2.39±0.55 | 2.40±0.58 | 2.38±0.52 |
| liver E | 17.93±7.88 | 18.27±8.32 | 17.50±7.28 |
| ATT | 0.58±0.11 | 0.55±0.10 | 0.61±0.11 |

MEAN, mean relative strain value within the ROI; SD, standard deviation of the relative strain value within the ROI); AREA, area of low strain within the ROI; COMP, complexity of low strain area within the ROI; KURT, kurtosis; SKEW, skewness; CONT, contrast; ENT, entropy; IDM, inverse difference moment; ASM, angular second moment; CORR, correlation; liver Vs: velocity of the shear wave; ATT, attenuation coefficient

**Table S4 Multivariate regression coefficients of 12 original dual elastography indices for EV classification in the training cohort**

|  | β Coefficient | P value |
| --- | --- | --- |
| MEAN | 0.019 | 0.394 |
| SD | 0.000 | 0.988 |
| AREA | 0.014 | 0.444 |
| COMP | 0.005 | 0.106 |
| KURT | -0.211 | 0.740 |
| SKEW | -0.815 | 0.487 |
| CONT | 0.002 | 0.391 |
| ENT | -1.480 | 0.378 |
| IDM | 1.751 | 0.049 |
| ASM | -9.718 | 0.653 |
| CORR | 12.489 | 0.523 |
| liver Vs | 0.353 | 0.018 |
| Intercept | -8.412 | 0.686 |

EV, esophageal varices; MEAN, mean relative strain value within the ROI; SD, standard deviation of the relative strain value within the ROI); AREA, area of low strain within the ROI; COMP, complexity of low strain area within the ROI; KURT, kurtosis; SKEW, skewness; CONT, contrast; ENT, entropy; IDM, inverse difference moment); ASM, angular second moment; CORR, correlation; liver Vs: velocity of the shear wave

**Table S5 Coefficients of three logistic regression models for predicting HRVs**

|  | Model 1 | Model 2 | Model 3 |
| --- | --- | --- | --- |
| PLT | -0.023 (-0.032 to -0.014) | -0.020 (-0.030 to -0.010) | -0.020 (-0.030 to -0.010) |
| ALT | -0.020 (-0.035 to -0.009) | -0.018 (-0.033 to -0.007) | -0.020 (-0.036 to -0.009) |
| ascites | - | 0.994 (0.277 to 1.727) | 0.734 (-0.060 to 1.538) |
| PVT | - | 0.894 (-0.031 to 1.892) | 0.789 (-0.030 to 0.010) |
| IDM | - | - | 4.041 (-0.880 to 11.895) |
| Liver Vs | - | - | 0.417 (-0.239 to 1.090) |
| Intercept | 2.586 (1.691 to 3.571) | 1.693 (0.698 to 2.755) | 0.337 (-1.569 to 2.254) |

HRVs: High-risk varices; PLT, platelet count; ALT, alanine aminotransferase; PVT: Portal vein thrombosis; IDM, inverse difference moment; IDM: inverse difference moment

Model 1 (LAB model): lab metrics (PLT+ALT)；Model 2 (LU model): lab metrics + ultrasonic indicators (PLT + ALT + ascites + PVT)；Model 3 (DELU model): lab metrics + ultrasonic indicators + dual-elasto parameters (PLT + ALT + ascites + PVT + IDM + liver Vs)

**Table S6 AUROCs of the DELU model and other NITs across subgroups in all patients with HBV-related cirrhosis**

|  | n | DELU model | Baveno VI | Expand Baveno VI | PSR | RESIST |
| --- | --- | --- | --- | --- | --- | --- |
| Total patients | 328 | 79.6 | 57.0 | 63.1 | 75.4 | 61.0 |
| Child-Pugh class |  |  |  |  |  |  |
| Class A | 274 | 79.7 | 57.6 | 64.5 | 77.0 | 62.5 |
| Class B+C | 54 | 80.3 | 54.2 | 56.7 | 66.9 | 54.2 |
| Anti-HBV treatment |  |  |  |  |  |  |
| Yes | 221 | 82.5 | 56.4 | 63.2 | 75.9 | 60.0 |
| No | 107 | 73.6 | 60.1 | 64.6 | 74.6 | 66.0 |
| Virally suppressed |  |  |  |  |  |  |
| Yes | 132 | 84.6 | 56.0 | 69.5 | 79.6 | 63.1 |
| No | 89 | 78.7 | 56.7 | 55.2 | 69.2 | 56.3 |
| Primary HCC |  |  |  |  |  |  |
| Yes | 89 | 81.8 | 57.7 | 71.1 | 77.3 | 65.7 |
| No | 239 | 78.7 | 56.7 | 60.0 | 74.7 | 59.2 |
| Male |  |  |  |  |  |  |
| Yes | 234 | 80.6 | 57.8 | 62.1 | 76.5 | 61.1 |
| No | 94 | 77.4 | 55.0 | 65.4 | 72.7 | 60.6 |
| BMI (kg/m^2^) |  |  |  |  |  |  |
| < 27 | 266 | 80.1 | 56.7 | 62.1 | 75.4 | 61.4 |
| ≥ 27 | 62 | 76.9 | 58.1 | 66.1 | 75.3 | 59.7 |

AUROC: Area Under the Receiver Operating Characteristic curve; NITs: noninvasive tests; HRVs：high-risk varices；HBV: hepatitis B virus；HCC: hepatocellular carcinoma; BMI: body mass index

**Table S7 Diagnostic performance of the DELU model and other NITs in Child-Pugh A and B/C subgroups**

|  | AUROC (%) | Sensitivity (%) | Specificity (%) | PPV (%) | NPV (%) | Spared EGD (%) | Missed HREV (%) |
| --- | --- | --- | --- | --- | --- | --- | --- |
| **Child Pugh A (n=274)** |  |  |  |  |  |  |  |
| DELU (cutoff 0.16) | 79.7 | 97.5 | 36.3 | 68.6 | 91.1 | 16.4 | 2.5 |
| Baveno VI | 57.6 | 99.4 | 15.8 | 62.4 | 94.7 | 6.9 | 0.6 |
| Expand Baveno VI | 64.5 | 91.3 | 37.7 | 67.3 | 75.4 | 20.8 | 8.8 |
| PSR | 77.0 | 92.5 | 40.4 | 68.5 | 79.3 | 21.2 | 7.5 |
| RESIST | 62.5 | 96.9 | 28.1 | 65.4 | 86.5 | 13.5 | 3.1 |
| **Child Pugh B+C (n=54)** |  |  |  |  |  |  |  |
| DELU (cutoff 0.16) | 80.3 | 96.6 | 32.0 | 62.2 | 88.9 | 16.7 | 3.4 |
| Baveno VI | 54.2 | 100 | 8.3 | 57.7 | 100 | 3.7 | 0 |
| Expand Baveno VI | 56.7 | 96.7 | 16.7 | 59.2 | 80.0 | 9.3 | 3.3 |
| PSR | 66.9 | 93.3 | 25.0 | 60.9 | 75.0 | 14.8 | 6.7 |
| RESIST | 54.2 | 100 | 8.3 | 57.7 | 100 | 3.7 | 0 |

NITs: noninvasive tests; HRVs：high-risk varices; AUROC: Area Under the Receiver Operating Characteristic curve; PPV, positive predictive value; NPV, negative predictive value；EGD: Esophagogastroduodenoscopy

**Table S8 Diagnostic performance of the DELU model and other NITs in ART-treated and ART-naïve subgroups**

|  | AUROC (%) | Sensitivity (%) | Specificity (%) | PPV (%) | NPV (%) | Spared EGD (%) | Missed HREV (%) |
| --- | --- | --- | --- | --- | --- | --- | --- |
| **ART (n=221)** |  |  |  |  |  |  |  |
| DELU (cutoff 0.16) | 82.5 | 98.2 | 35.5 | 60.6 | 95.1 | 18.6 | 1.8 |
| Baveno VI | 56.4 | 100 | 12.7 | 53.6 | 100 | 6.3 | 0 |
| Expand Baveno VI | 63.2 | 93.7 | 32.7 | 58.4 | 83.7 | 19.5 | 6.3 |
| PSR | 75.9 | 92.8 | 34.5 | 58.9 | 82.6 | 20.8 | 7.2 |
| RESIST | 60.0 | 98.2 | 21.8 | 55.9 | 92.3 | 11.8 | 1.8 |
| **Naive-ART (n=107)** |  |  |  |  |  |  |  |
| DELU (cutoff 0.16) | 74.3 | 96.2 | 35.7 | 80.9 | 76.9 | 12.1 | 3.8 |
| Baveno VI | 60.1 | 98.7 | 21.4 | 78.0 | 85.7 | 6.5 | 1.3 |
| Expand Baveno VI | 64.6 | 89.9 | 39.3 | 80.7 | 57.9 | 17.8 | 10.1 |
| PSR | 74.6 | 92.4 | 50.0 | 83.9 | 70.0 | 18.7 | 7.6 |
| RESIST | 66.0 | 96.2 | 35.7 | 80.9 | 76.9 | 12.1 | 3.8 |

NITs: noninvasive tests; HRVs：high-risk varices; HBV: hepatitis B virus；ART: antiviral therapy; AUROC: Area Under the Receiver Operating Characteristic curve; PPV, positive predictive value; NPV, negative predictive value；EGD: Esophagogastroduodenoscopy

**Table S9 Diagnostic performance of the DELU model and other NITs in virally suppressed and non-suppressed ART subgroups**

|  | AUROC (%) | Sensitivity (%) | Specificity (%) | PPV (%) | NPV (%) | Spared EGD (%) | Missed HREV (%) |
| --- | --- | --- | --- | --- | --- | --- | --- |
| **Virally suppressed (n=132)** |  |  |  |  |  |  |  |
| DELU (cutoff 0.16) | 84.6 | 98.7 | 29.8 | 64.9 | 94.4 | 13.6 | 1.3 |
| Baveno VI | 56.0 | 100 | 12.1 | 59.2 | 100 | 5.3 | 0 |
| Expand Baveno VI | 69.5 | 95.9 | 43.1 | 68.3 | 89.3 | 21.2 | 4.1 |
| PSR | 79.6 | 94.6 | 37.9 | 66.0 | 84.6 | 19.7 | 5.4 |
| RESIST | 63.1 | 98.6 | 27.6 | 63.5 | 94.1 | 12.9 | 1.4 |
| **Non-suppressed (n=89)** |  |  |  |  |  |  |  |
| Model CEI (cutoff 0.16) | 78.7 | 97.2 | 41.5 | 53.0 | 95.7 | 25.8 | 2.8 |
| Baveno VI | 56.7 | 100 | 13.5 | 45.1 | 100 | 7.9 | 0 |
| Expand Baveno VI | 55.2 | 89.2 | 21.2 | 44.6 | 73.3 | 16.9 | 10.8 |
| PSR | 69.2 | 89.2 | 30.8 | 47.8 | 80.0 | 22.5 | 10.8 |
| RESIST | 56.3 | 97.3 | 15.4 | 45.0 | 88.9 | 10.1 | 2.7 |

NITs: noninvasive tests; HRVs：high-risk varices; AUROC: Area Under the Receiver Operating Characteristic curve; PPV, positive predictive value; NPV, negative predictive value；EGD: Esophagogastroduodenoscopy

**Table S10 Diagnostic performance of the DELU model and other NITs in primary HCC and non-HCC subgroups**

|  | AUROC (%) | Sensitivity (%) | Specificity (%) | PPV (%) | NPV (%) | Spared EGD (%) | Missed HREV (%) |
| --- | --- | --- | --- | --- | --- | --- | --- |
| **Primary HCC (n=89)** |  |  |  |  |  |  |  |
| DELU (cutoff 0.16) | 81.8 | 98.0 | 46.2 | 70.0 | 94.7 | 21.3 | 2.0 |
| Baveno VI | 57.7 | 100 | 15.4 | 60.2 | 100 | 6.7 | 0 |
| Expand Baveno VI | 71.1 | 96.0 | 46.2 | 69.6 | 90.0 | 22.5 | 4.0 |
| PSR | 77.3 | 94.0 | 46.2 | 69.1 | 85.7 | 23.6 | 6.0 |
| RESIST | 65.7 | 98.0 | 33.3 | 65.3 | 92.9 | 15.7 | 2.0 |
| **Non-Primary HCC (n=239)** |  |  |  |  |  |  |  |
| DELU (cutoff 0.16) | 78.7 | 97.1 | 31.3 | 66.7 | 88.6 | 14.6 | 2.9 |
| Baveno VI | 56.7 | 99.3 | 14.1 | 62.1 | 93.3 | 6.3 | 0.7 |
| Expand Baveno VI | 60.0 | 90.7 | 29.3 | 64.5 | 69.0 | 17.6 | 9.3 |
| PSR | 74.7 | 92.1 | 34.3 | 66.5 | 75.6 | 18.8 | 7.9 |
| RESIST | 59.2 | 97.1 | 21.2 | 63.6 | 84.0 | 10.5 | 2.9 |

NITs: noninvasive tests; HRVs：high-risk varices; HCC: hepatocellular carcinoma; HBV: hepatitis B virus；AUROC: Area Under the Receiver Operating Characteristic curve; PPV, positive predictive value; NPV, negative predictive value；EGD: Esophagogastroduodenoscopy

**Table S11 Diagnostic performance of the DELU model and other NITs in male and female subgroups**

|  | AUROC (%) | Sensitivity (%) | Specificity (%) | PPV (%) | NPV (%) | Spared EGD (%) | Missed HREV (%) |
| --- | --- | --- | --- | --- | --- | --- | --- |
| **Male (n=234)** |  |  |  |  |  |  |  |
| DELU (cutoff 0.16) | 80.6 | 97.1 | 37.8 | 68.4 | 90.2 | 17.5 | 2.9 |
| Baveno VI | 57.8 | 99.3 | 16.3 | 62.2 | 94.1 | 7.3 | 0.7 |
| Expand Baveno VI | 62.1 | 92.6 | 31.6 | 65.3 | 75.6 | 17.5 | 7.4 |
| PSR | 76.5 | 93.4 | 38.8 | 67.9 | 80.9 | 20.1 | 6.6 |
| RESIST | 61.1 | 97.8 | 24.5 | 64.3 | 88.9 | 11.5 | 2.2 |
| **Female (n=94)** |  |  |  |  |  |  |  |
| DELU (cutoff 0.16) | 77.4 | 98.1 | 30.0 | 65.4 | 92.3 | 13.8 | 1.9 |
| Baveno VI | 55.0 | 100 | 10.0 | 60.0 | 100 | 4.3 | 0 |
| Expand Baveno VI | 65.4 | 90.7 | 40.0 | 67.1 | 76.2 | 22.3 | 9.3 |
| PSR | 72.7 | 90.7 | 35.0 | 65.3 | 73.7 | 20.2 | 9.3 |
| RESIST | 60.6 | 96.3 | 25.0 | 63.4 | 83.3 | 12.8 | 3.7 |

NITs: noninvasive tests; HRVs：high-risk varices; HBV: hepatitis B virus；AUROC: Area Under the Receiver Operating Characteristic curve; PPV, positive predictive value; NPV, negative predictive value；EGD: Esophagogastroduodenoscopy

**Table S12 Diagnostic performance of the DELU model and other NITs in patients with BMI < 27 and BMI ≥ 27**

|  | AUROC (%) | Sensitivity (%) | Specificity (%) | PPV (%) | NPV (%) | Spared EGD (%) | Missed HREV (%) |
| --- | --- | --- | --- | --- | --- | --- | --- |
| **BMI < 27 kg/m^2^ (n=266)** |  |  |  |  |  |  |  |
| DELU (cutoff 0.16) | 80.1 | 97.5 | 36.4 | 69.5 | 90.7 | 16.2 | 2.5 |
| Baveno VI | 56.7 | 99.4 | 14.0 | 63.2 | 93.8 | 6.0 | 0.6 |
| Expand Baveno VI | 62.1 | 92.5 | 31.8 | 66.8 | 73.9 | 17.3 | 7.5 |
| PSR | 75.4 | 92.5 | 36.4 | 68.4 | 76.5 | 19.2 | 7.5 |
| RESIST | 61.4 | 97.5 | 25.2 | 66.0 | 87.1 | 11.7 | 2.5 |
| **BMI ≥ 27 kg/m^2^ (n=62)** |  |  |  |  |  |  |  |
| DELU (cutoff 0.16) | 76.9 | 96.8 | 32.3 | 58.8 | 90.9 | 17.7 | 3.2 |
| Baveno VI | 58.1 | 100 | 16.1 | 54.4 | 100 | 8.1 | 0 |
| Expand Baveno VI | 66.1 | 90.3 | 41.9 | 60.9 | 81.3 | 25.8 | 9.7 |
| PSR | 75.3 | 93.5 | 41.9 | 61.7 | 86.7 | 24.2 | 6.5 |
| RESIST | 59.7 | 96.8 | 22.6 | 55.6 | 87.5 | 12.9 | 3.2 |

NITs: noninvasive tests; HRVs：high-risk varices; BMI: body mass index; HBV: hepatitis B virus；AUROC: Area Under the Receiver Operating Characteristic curve; PPV, positive predictive value; NPV, negative predictive value；EGD: Esophagogastroduodenoscopy

**Table S13 Diagnostic performance of the DELU model and other NITs in patients with LSM ≥ 10 kPa and LSM < 10 kPa**

|  | AUROC (%) | Sensitivity (%) | Specificity (%) | PPV (%) | NPV (%) | Spared EGD (%) | Missed HREV (%) |
| --- | --- | --- | --- | --- | --- | --- | --- |
| **LSM ≥ 10 kPa (n=269)** |  |  |  |  |  |  |  |
| DELU (cutoff 0.16) | 75.9 | 97.0 | 27.6 | 67.7 | 85.3 | 12.6 | 3.0 |
| Baveno VI | 54.0 | 99.4 | 8.6 | 62.9 | 90.0 | 3.7 | 0.6 |
| Expand Baveno VI | 57.9 | 92.1 | 23.8 | 65.4 | 65.8 | 14.1 | 7.9 |
| PSR | 70.9 | 92.1 | 29.5 | 67.1 | 70.5 | 16.4 | 7.9 |
| RESIST | 56.9 | 97.6 | 16.2 | 64.5 | 81.0 | 7.8 | 2.4 |
| **LSM < 10 kPa (n=59)** |  |  |  |  |  |  |  |
| DELU (cutoff 0.16) | 94.2 | 100 | 60.6 | 66.7 | 100 | 33.9 | 0 |
| Baveno VI | 66.7 | 100 | 33.3 | 54.2 | 100 | 18.6 | 0 |
| Expand Baveno VI | 79.5 | 92.3 | 66.7 | 68.6 | 91.7 | 40.7 | 7.7 |
| PSR | 88.8 | 96.2 | 63.6 | 67.6 | 95.5 | 37.3 | 3.8 |
| RESIST | 73.8 | 96.2 | 51.5 | 61.0 | 94.4 | 30.5 | 3.8 |

NITs: noninvasive tests; HRVs：high-risk varices; LSM: liver stiffness measurement; HBV: hepatitis B virus；AUROC: Area Under the Receiver Operating Characteristic curve; PPV, positive predictive value; NPV, negative predictive value；EGD: Esophagogastroduodenoscopy

**Supplementary Figures**

**Figure S1 Schematic diagram of dual-elastography imaging operation**


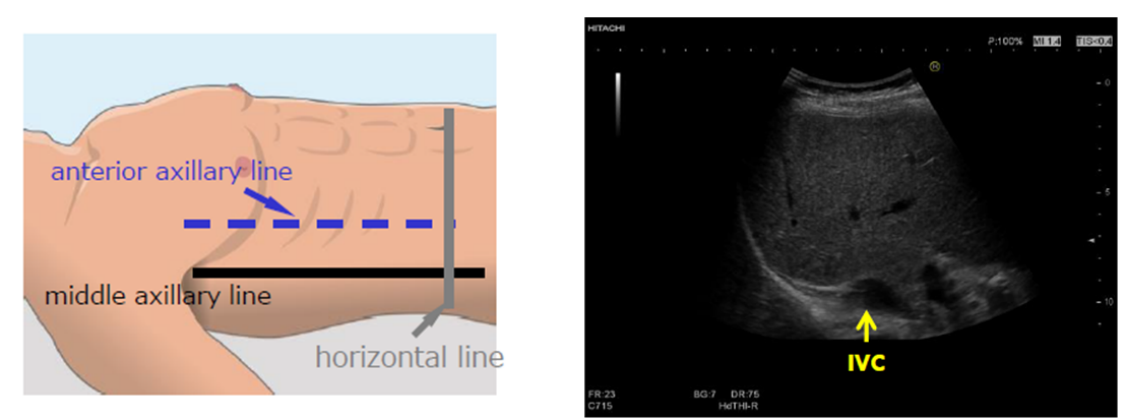


1. (b)

On the left toolbar of the touchscreen, select the Combi-Elasto mode. The patient should be positioned in a horizontal supine posture (if the image quality is suboptimal, a left lateral decubitus position can be considered). The operator should place the probe perpendicular to the liver's capsule through the right intercostal space, carefully avoiding large blood vessels. The measurement depth should be set between 1-2 cm, though it is advisable not to exceed 5 cm. The sampling frame, which is pre-configured and does not require further adjustment, should be placed in the right lobe of the liver (segment S5 or S8). Ensure that the inferior vena cava (IVC) remains centered in the image.

**Figure S2 Calibration curves of the DELU model in the (a) training and (b) validation cohorts**


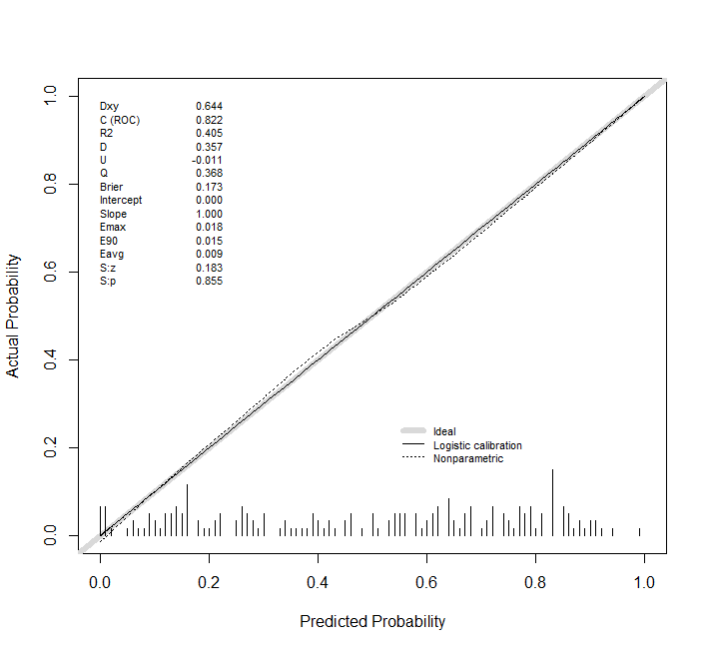

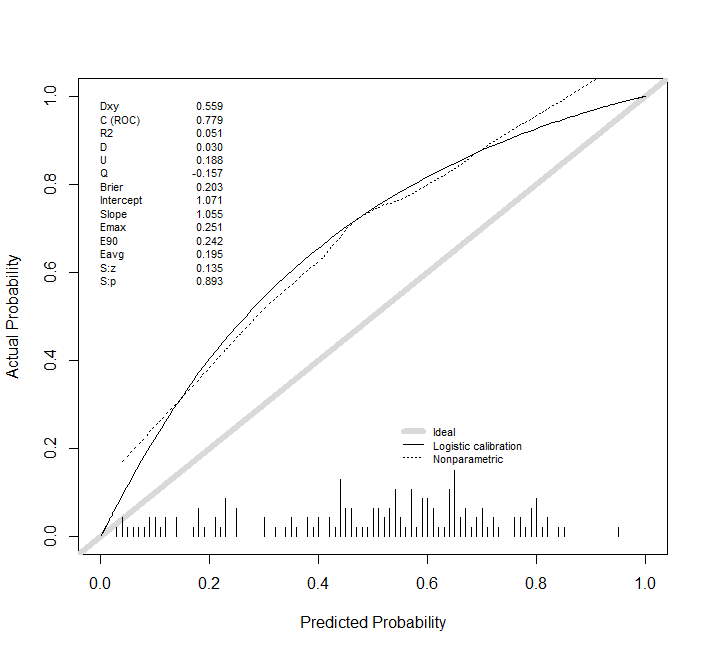


(a) (b)

Good calibration curves were observed in both the training and validation cohorts (*p* > 0.05).

**Figure S3 Decision curve analysis (DCA) of the DELU model in the (a) training and (b) validation cohorts**


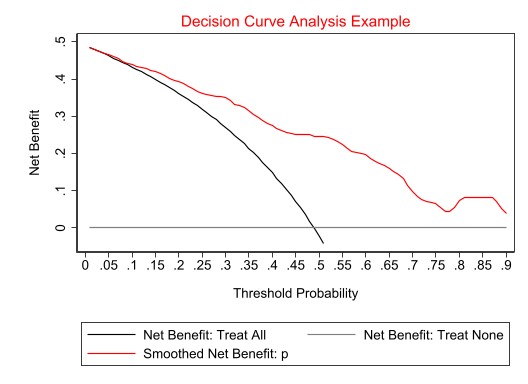

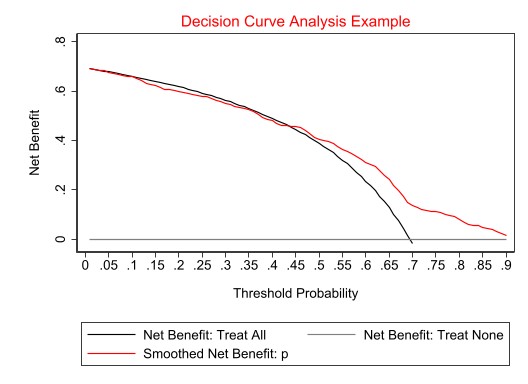


1. (b)

The decision curve analysis (DCA) showed the DELU nomogram had a wide threshold probability range, indicating a valuable net benefit for predicting HRV in HBV-related cirrhosis.
